# A comparative study of some NSAIDs with natural products: integrating in vitro anticancer efficacy, in vivo antiulcerative effect, histochemistry, and in silico analysis

**DOI:** 10.1007/s00210-026-05041-1

**Published:** 2026-02-04

**Authors:** Rabia Selina Hal, Prestige Vialli Moyo, Kadircan Ural, Merve Sıkık, Ayla Nur Demiral, Mehmet Akif Ovali, Alper Onder, Neslihan Kaya Terzi, Berrin Erkan, Ferah Comert Onder

**Affiliations:** 1 Basic Medical Science, Faculty of Medicine, Çanakkale Onsekiz Mart University, 17020 Çanakkale, Turkey; 2 Faculty of Medicine, University of Münster, 48149 Münster, Germany; 3 Department of Medical System Biology, School of Graduate Students, Çanakkale Onsekiz Mart University, 17020 Çanakkale, Turkey; 4 Department of Physiology, Faculty of Medicine, Çanakkale Onsekiz Mart University, 17020 Çanakkale, Turkey; 5 Natural Product and Drug Research Laboratory, Department of Chemistry, Faculty of Science, Çanakkale Onsekiz Mart University, 17020 Çanakkale, Turkey; 6 Department of Pathology, Faculty of Medicine, Çanakkale Onsekiz Mart University, 17020 Çanakkale, Turkey; 7 Department of Statistics, Faculty of Science, Çanakkale Onsekiz Mart University, 17020 Çanakkale, Turkey; 8 Department of Medical Biology, Faculty of Medicine, Çanakkale Onsekiz Mart University, 17020 Çanakkale, Turkey

**Keywords:** Celecoxib, Natural products, Anticancer, In vivo antiulcer, MD simulation

## Abstract

Assessing the biological activities of some potential drugs and comparing their suitability for in vitro and in vivo combination therapy or in silico drug repositioning against important targets is essential for minimizing labor, costs, and time in drug development. Herein, dose- and time-dependent in vitro anticancer activity studies of anti-inflammatory drugs, including celecoxib (**C**), indomethacin (**I**), and meloxicam (**M**), in combination with natural products (taxifolin (**T**), quercetin (**Q**), and rutin (**R**)) and doxorubicin (Dox), were carried out in MDA-MB-231, BT-20, MCF-7, and HT-29 human cancer cell lines. Drug **C** demonstrated significant anticancer activity in cancer cells with natural products (< 40 µM) and Dox (< 5 µM). The antiulcerative effect of the most promising drug **C** in combination with **T** and **R** in rats was carried out. The histopathological analysis suggests that the substitution of **R** with **T**, when combined with drug **C**, leads to a statistically significant improvement in the amelioration of gastric mucosal injury. Additionally, in silico studies have been conducted against the important cancer drug target sphingosine kinase 1 (SphK1). The obtained results highlight that drug **C** and **T** may be potential inhibitor candidates as SphK1 inhibitors for targeted cancer therapy. Overall, the combination of drug **C** with **T** has shown promising results, anticancer effects in breast and colon cancer cells and antiulcerative effects in rats.

## Introduction

The natural compounds derived from plants, also referred to as secondary metabolites such as flavonoids, alkaloids, tannins, and terpenoids, have demonstrated numerous effects on human health. Natural compounds have served as valuable resources for the treatment of diseases including neurodegenerative diseases, cancer, ulcerative colitis, diabetes, cardiovascular diseases, digestive system disorders, inflammation, and aging (Fatima et al. 2025). The combination therapy involving natural compounds has offered greater effectiveness compared to single-agent treatments. Its aim was to maximize efficacy while minimizing possible side effects in combination therapies (Sauter 2020). Moreover, combination therapies have played crucial roles in the treatment of several diseases, especially in cancer treatment, by involving the use of two or more therapeutic agents to fight the disease (Mokhtari et al. 2017). This approach has been essential due to the limitations of single-agent therapies, which have often been compromised by tumor heterogeneity (Janku et al. 2014). Drug combinations have made these agents reusable by targeting alternative signaling pathways associated with specific cancer characteristics, therefore sensitizing cancer cells to other cytotoxic substances (Zhang et al. 2020). The most preferred flavonoids in combination therapy are polyphenolic compounds that can be used as potential therapeutic agents. Among the flavonoids, quercetin (**Q**) and rutin (**R)** have been of interest to researchers. Today, the medical use of natural product **Q** is explained by its broad-spectrum biological properties such as antioxidant, anti-inflammatory, antiplatelet, and anti-apoptotic (Salehi et al. 2020; Satari et al. 2021).

Ulcer disease is one of the most significant and serious gastrointestinal disorders. Peptic ulcers encompassed both gastric/stomach and duodenum ulcers (Lanas and Chan 2017). The gastric mucosa is compromised by the effects of elevated gastric acid, pepsin, bacterial infection, and chronic use of non-steroidal anti-inflammatory drugs (Drini 2017). It is reported that the global prevalence of peptic ulcers ranges between 5 and 10% (Lanas and Chan 2017). Long-term use of NSAIDs inhibits the synthesis of prostaglandins (PG), which have vital roles in the gastrointestinal tract. PG, produced from arachidonic acid by cyclooxygenase (COX) enzymes, plays vital roles in maintaining the gastric mucosa (Sohail et al. 2023). Furthermore, prostaglandins regulate motility, exert cytoprotective and secretory effects, inhibit acid secretion, stimulate bicarbonate production, and enhance mucosal blood flow (Miller 1983).

COX enzymes (COX-1 and COX-2) have several important roles in cardiovascular, neuronal, renal, gastrointestinal, immune, and reproductive systems, which produce prostaglandins, thromboxane, and levuloglandins (Fitzpatrick 2004). COX-1 is essential for the gastrointestinal mucosa and vascular homeostasis (Husdal et al. 2006). COX-2 is overexpressed in malignant and metastatic epithelial tumors (Singh et al. 2010). The increased expression level of COX-2 is known as a poor prognosis as it promotes cell proliferation, invasion, and metastasis in carcinogenesis. COX-2 shows its effects as a rate-limiting enzyme in PG synthesis. There are several COX inhibitors including aspirin, meloxicam, indomethacin, piroxicam, and celecoxib which are the most well-known NSAIDs (Ahmadi et al. 2022; Grosch et al. 2006). Additionally, the inhibition of the COX enzyme with NSAIDs also causes gastrointestinal diseases such as gastric mucosa injury and ulcer (Takeuchi et al. 2005). Celecoxib (**C**), one of the NSAIDs, is a non-addictive, selective COX-2 inhibitor with analgesic, antipyretic, anti-inflammatory, anticarcinogenic, and cartilage-protective properties. Thus, its main mechanism of action is to inhibit the COX enzyme responsible for prostaglandin production. COX-2 plays an important role in cancer cell growth and activates the angiogenic pathway by adjusting vascular endothelial growth factor (VEGF) levels. Indomethacin (**I**) is an NSAID that has been extensively studied for various medical applications, including pancreatitis (He et al. 2018), ophthalmic use (Toropainen et al. 2021), corneal penetration (Firozian et al. 2023), gastrointestinal effects (Muñoz-Miralles et al. 2018), cancer treatment (Liu et al. 2021), pharmacokinetics (Pillai et al. 2021), and headache disorders (Farag and Bahra 2022). Additionally, drug **I** is a nonselective COX inhibitor and inhibits the synthesis of prostaglandin receptor (PGE2) (Pacifici 2013; Lucas 2016). Meloxicam (**M**), a selective COX-2 inhibitor, has a potential therapeutic effect against hepatocellular carcinoma (HCC) cells (Dong et al. 2014). Also, according to its inhibitory roles of COX-2 enzyme, meloxicam can cause mucosal injury and bleeding.

More than 90% of new drug candidates fail during drug development. However, drug repositioning has been used as an important strategy to identify new clinical applications for approved drugs, increasing the potential use of existing drugs (Liao et al. 2012). Zidovudine and Raloxifene are examples of drugs whose secondary effects have been successfully determined by drug repositioning to date (Pushpakom et al. 2019). In conclusion, while cancer remains a major global health problem, drug repurposing offers several advantages over the development of new compounds, such as lower cost and better safety. COX-2 inhibitors are one of the most promising drug classes for repurposing in cancer therapy.

Therefore, this study focused on the anticancer effects of the widely used NSAID drugs (**C, I**, and **M)** on various human breast and colon cancer cell lines, both alone and in combination with natural products (**Q**, **R**, and **T**) and a chemotherapeutic drug doxorubicin (Dox). Moreover, the combination of the promising drug **C** with natural products was investigated for its antiulcer effect in rats. To the best of our knowledge, this is the first study to explore the anticancer and antiulcerative effects of drug** C** through both in vitro and in vivo combination studies with natural compounds and in silico analyses against important cancer drug target, sphingosine kinase 1 (SphK1). As a result, drug **C** could be suggested as a promising agent with its in vitro anticancer effect in combination with Dox and in vivo antiulcer effect in combination with natural product (**T**).

## Materials and methods

### In vitro anticancer study

#### Cell culture and reagents

Human triple negative breast cancer (TNBC) (MDA-MB-231 and BT-20), estrogen receptor positive (ER +) (MCF-7), and colon (HT-29) cancer cell lines were gifted and cultured in Dulbecco’s modified Eagle medium (DMEM) containing 2 mM L-glutamine, 10% fetal bovine serum (FBS), and 1% penicillin/streptomycin (PS). The cultures were maintained in a 37 °C incubator with 5% CO_2_. All experiments were conducted with multiple replicates (Comert Onder et al. 2025; Onder et al. 2023).

#### Cytotoxicity assay

To determine the cytotoxic effects of the drugs (**C**, **I**, and **M)** on various human cancer cell lines, MTT (3-(4,5-dimethyl-2-thiazolyl)−2,5-diphenyl tetrazolium bromide) was used. The cells were seeded in a 96-well plate, and the drugs were applied to the cells alone and in combination with the natural products **Q**, **R**, **T**, and a chemotherapeutic drug Dox in increasing doses for 72 h. DMSO was used as a control. The absorbances were measured at 570 nm in a microplate reader (Comert Onder et al. 2025; Onder et al. 2023).

### In vivo antiulcer study

#### Study design

This study was conducted using 28 male Wistar rats, aged 2–3 months and weighing 200–300 g, obtained from the Experimental Research Application and Research Center of Çanakkale Onsekiz Mart University (ÇOMUDAM). Throughout the study period, the rats were housed at the ÇOMUDAM facility under controlled environmental conditions and a 12-h light/12-h dark cycle. All animals were fed ad libitum. Rats in the groups subjected to ulcer induction were fasted for 24 h prior to the procedure, with free access to water.

#### Experimental groups

Only healthy 2–3-month-old rats were included in the study. The animals were randomly assigned into the following groups:

- **Control group (*n* = 7):** No treatment was administered to the rats in this group.
- **Ulcer group (*n* = 7):** After 24 h of fasting (with free access to water), rats received 1 mL of absolute ethanol (> 99.5%) per rat via oral gavage to induce gastric ulceration. One hour (60 min) post-administration, the entire stomach tissue was collected.
- **Ulcer + celecoxib + rutin group (*n* = 7):** Following 24 h of fasting, rats in this group were administered celecoxib (5 mg/kg, intraperitoneally) and rutin (80 mg/kg, orally via gavage) as antiulcer agents. After 1 h, 1 mL of absolute ethanol (> 99.5%) was administered orally. One hour after ethanol exposure, stomach tissues were collected.
- **Ulcer + celecoxib + taxifolin group (*n* = 7):** After 24 h of fasting, the rats received celecoxib (5 mg/kg, intraperitoneally) and taxifolin (50 mg/kg, dissolved in distilled water) via oral gavage, as described by Olaleye and Akinmoladun (2013). One hour later, 1 mL of absolute ethanol (> 99.5%) was administered orally. After a further 60-min period, the stomach tissues were harvested. All the tissues were harvested under general anesthesia (70 mg/kg/7 mg/kg ketamin/xylazine, i.m.).

### Histochemistry

Representative tissue specimens were fixed in 10% neutral buffered formalin and subsequently embedded in paraffin to prepare histological blocks. Serial sections of 4-µm thickness were obtained using a rotary microtome and mounted onto glass slides. These sections were stained with hematoxylin and eosin (H&E) according to standard histological protocols and examined using a light microscope (Olympus BX46). Mason trichrome stain was applied for fibrosis evaluation. Histopathological evaluation was performed by an experienced pathologist, and the following parameters were evaluated: epithelial cell loss, bleeding, edema, inflammatory cell infiltration (mononuclear cells and neutrophils), mucosal erosion/ulcer, and fibrosis (Minaiyan et al. 2018). The epithelial defect score represented the gastric injury, with the scoring system according to Roger’s criteria (Rogers 2012).

The extent of gastric injury was evaluated using the Histological Activity Index (HAI) method as described by Rogers (2012), with modifications to reflect the degree of tissue vulnerability. Scoring was conducted on a scale from 0 to 4, where 0 indicates no abnormality across the entire microscopic field of view; 1 represents abnormalities affecting less than 25% of the field; 2 corresponds to abnormalities involving 25%–50% of the field; 3 indicates 50%–75% involvement; and 4 reflects abnormalities present in more than 75% of the entire field of view. All scores were given based on the average of the ratings of ten areas under the microscope, and scoring evaluation was determined semi-quantitatively. Sample light microscopic images from the study groups are shown in Fig. 5.

### Statistical analysis

Statistical analysis was performed by using GraphPad Prism software, and significant results of *p* values were evaluated for in vitro analysis.

For in vivo analysis, the Kruskal–Wallis H Test was used to determine the differences between groups among non-parametric tests. The results were examined at a significance level of *α* = 0.05, and *p* < 0.05 was considered statistically significant. For findings with statistically significant differences, the Bonferroni test, a post hoc test, was used to determine which groups revealed significance. All analysis results were obtained with the IBM-SPSS 27 program.

### In silico analysis

Glide/SP method (Schrödinger Release LLC, New York, NY) was used for molecular docking study (Friesner et al. 2006). The crystal structure of sphingosine kinase 1 (SphK1) was downloaded from Protein Data Bank (PDB) (pdb: 3VZB) (https://www.rcsb.org/). Protein Preparation Wizard and LipPrep were used (Sastry et al. 2013). PROPKA and OPLS4, respectively, were used for optimization and minimization of the target protein and ligands (Lu et al. 2021). Epik was used to predict pKa and protonation state distributions (Johnston et al. 2023). Ionization state was generated at target pH 7. Maximum ligand size was selected for 500 atoms and generated at most 32 per ligand by LigPrep. Molecular dynamics (MD) simulations for 500 ns were performed using Desmond of Maestro (Bowers et al. 2006; Kalin and Comert Onder 2025). Top docking poses of the drugs were used. The OPLS4 force field was used. The simulations were conducted at a constant temperature of 300 K and a pressure of 1 atm. The TIP3P water model was used to solve the systems, and counter ions Na + and Cl − (0.15 M) were added to neutralize the system’s charge. Box shape was selected as orthorhombic. Nose–Hoover thermostat and Martyna–Tobias–Klein barostat methods were used. The root-mean-square deviation (RMSD) and the root-mean-square fluctuation (RMSF) were analyzed.

## Results

### In vitro anticancer study

**Fig. 1:**
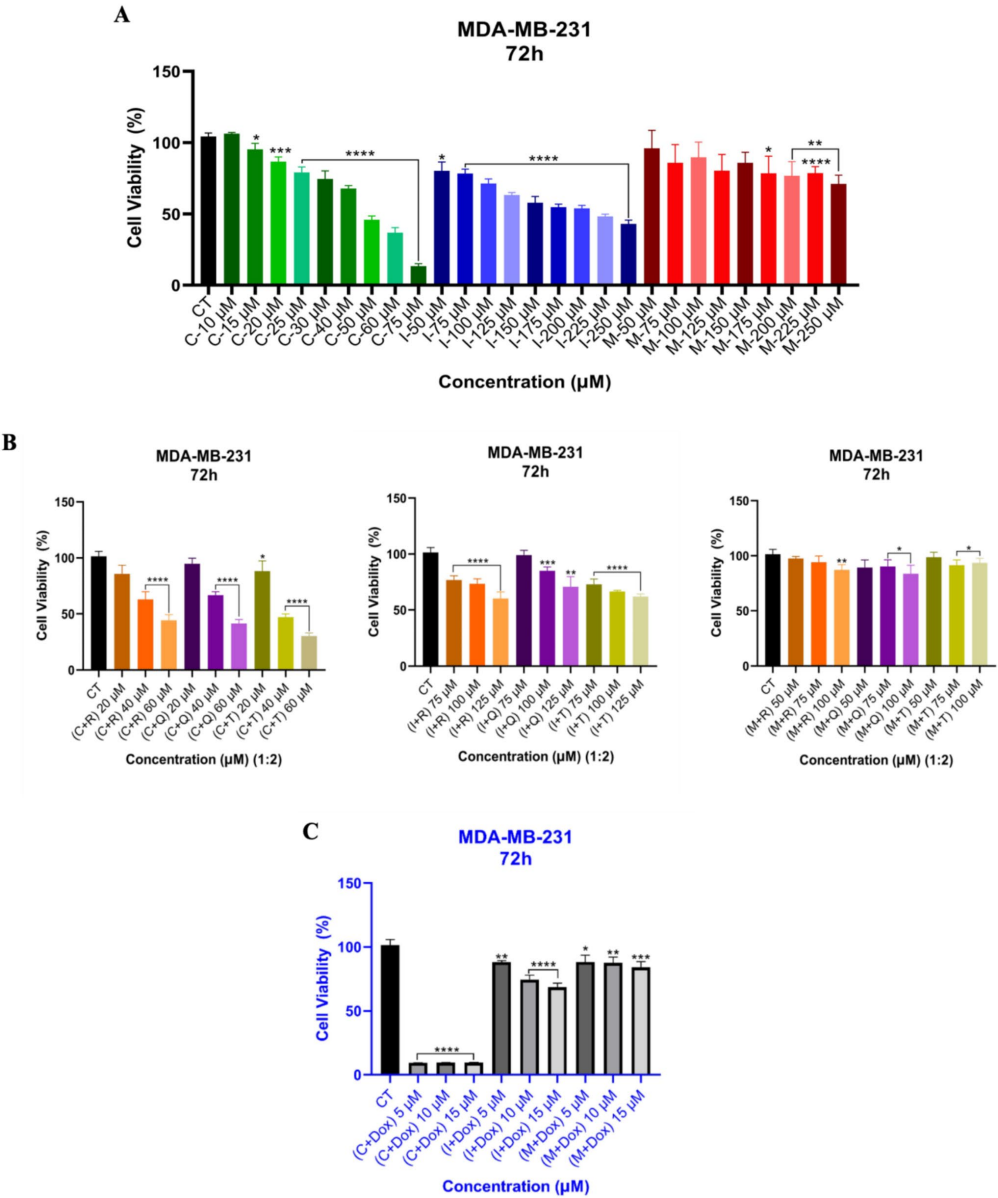
The cytotoxicity of drugs alone (**a**) and in combination with natural products (**R, Q**, and **T**), 20 µM, 40 µM, and 60 µM for drug **C**; 75 µM, 100 µM, and 125 µM for drug **I**; and 50 µM, 75 µM, and 100 µM for drug **M**. The 1:2 ratio represents drug (1) to natural product (2). **R** (20 µM), **Q** (10 µM), and **T** (10 µM) (**b**) and Dox (0.05 µM) (**c**) against MDA-MB-231 TNBC cells. ****p value < 0.0001, ***p value < 0.0002

To determine the inhibition profiles of the studied NSAID drugs on human cancer cell lines, a cytotoxicity assay was performed for the treatments with each drug alone and their combined treatments with natural products (**Q, R**, and **T**) and a well-known chemotherapeutic drug Dox. As seen in Fig. 1a, drug **C** exhibited significant cell cytotoxicity in MDA-MB-231 cells between 50 and 75 µM for 72-h treatments, whereas drugs **I** and **M** alone did not inhibit the cell cytotoxicity up to 250 µM. The inhibition profile of drug **C** was examined with the combination of **T** in MDA-MB-231 cells; the cell viability was dramatically decreased at 40 µM compared to the combination with **R** and **Q** in the increasing concentrations (Fig. 1b). The combination of drugs **I** and **M** with natural products did not exhibit a significant inhibition profile on the cancer cell line (Fig. 1b). Furthermore, when Dox concentration was kept constant (0.05 µM) for the combination of drug** C** in the increasing concentrations (5, 10, and 15 µM), the cell viability was excessively decreased (Fig. 1c). These findings show that drug **C** in alone and the combination with a natural product (**T**) has anticancer activity in MDA-MB-231 cells, and it can be used at lower concentrations to reduce the side effects of Dox for chemotherapy.

**Fig. 2:**
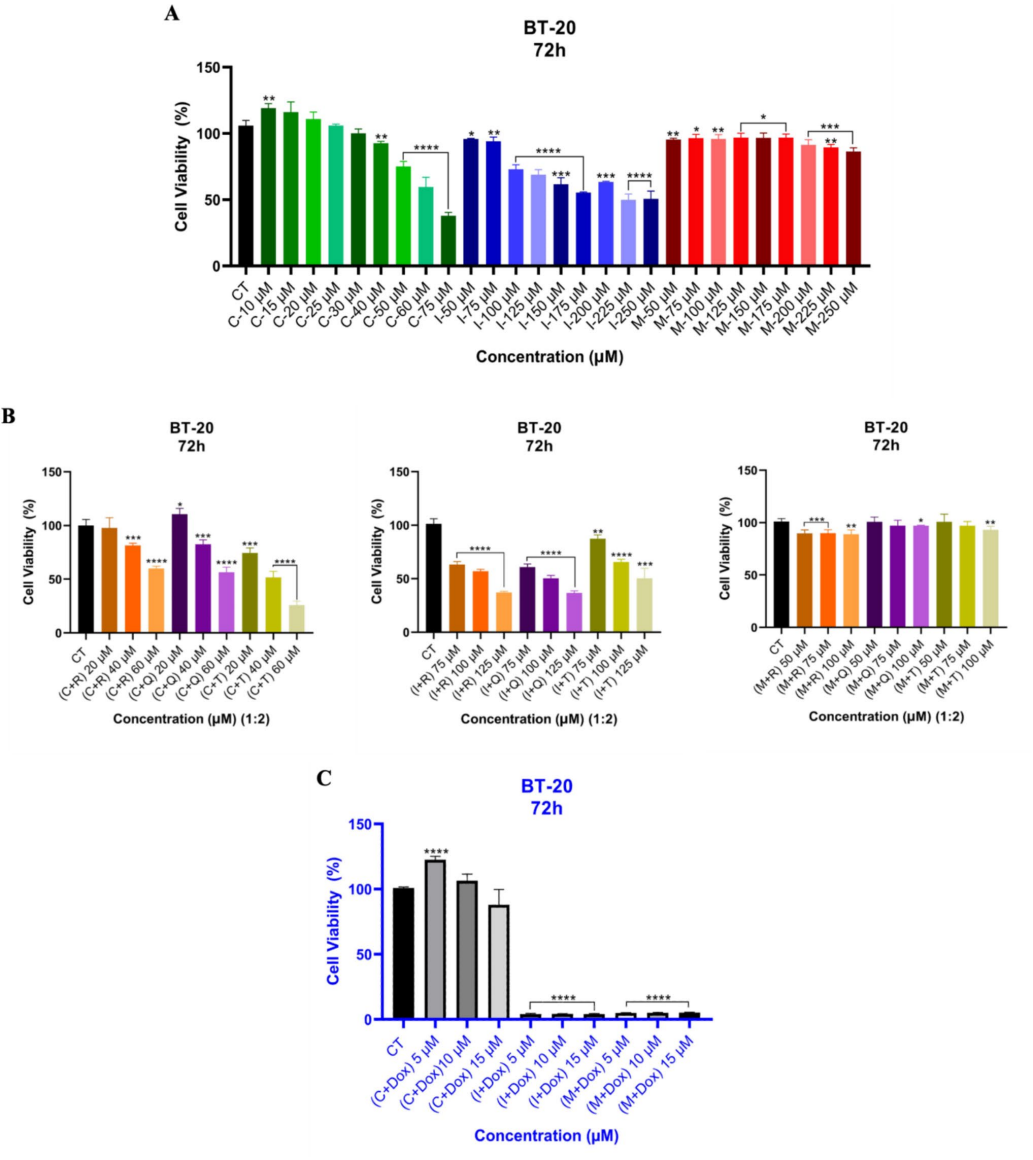
The cytotoxicity of drugs in isolation (**a**) and the combination with natural products (**R, Q**, and **T**), 20 µM, 40 µM, and 60 µM for drug **C**; 75 µM, 100 µM, and 125 µM for drug **I**; and 50 µM, 75 µM, and 100 µM for drug **M**. The 1:2 ratio represents drug (1) to natural product (2). **R** (20 µM), **Q** (10 µM), and **T** (10 µM) (**b**) and Dox (0.05 µM) (**c**) against BT-20 TNBC cells. ****p value < 0.0001, ***p value < 0.0002

As seen in Fig. 2a, drug **C** exhibited significant cell cytotoxicity on BT-20 cells at approximately 75 µM for 72-h treatment, whereas drugs **I** and **M** alone did not inhibit the cytotoxicity up to 225 µM. However, the combination of drug **C** with **T** showed an inhibition effect at a 40 µM concentration. Although drug **I** alone inhibited the cell viability at a 250 µM concentration, the combined treatment with **R**, **Q**, and **T**, respectively, resulted in the suppression of cell viability at 125 µM for each treatment (Fig. 2b). Furthermore, each drug was studied with the combination at a constant concentration of Dox (0.05 µM), and drugs **I** and **M** showed significant inhibition on the cytotoxicity (Fig. 2c). These findings show that drugs **C** and** I** in combination with natural products (**R, Q**, and **T**) have anticancer activity on BT-20 TNBC cells, and drugs **I** and **M** can be used at lower concentrations to reduce the side effects of Dox for chemotherapy. On the other hand, drug **M** was not as effective as the other drugs and their combinations (Fig. 2b).

**Fig. 3:**
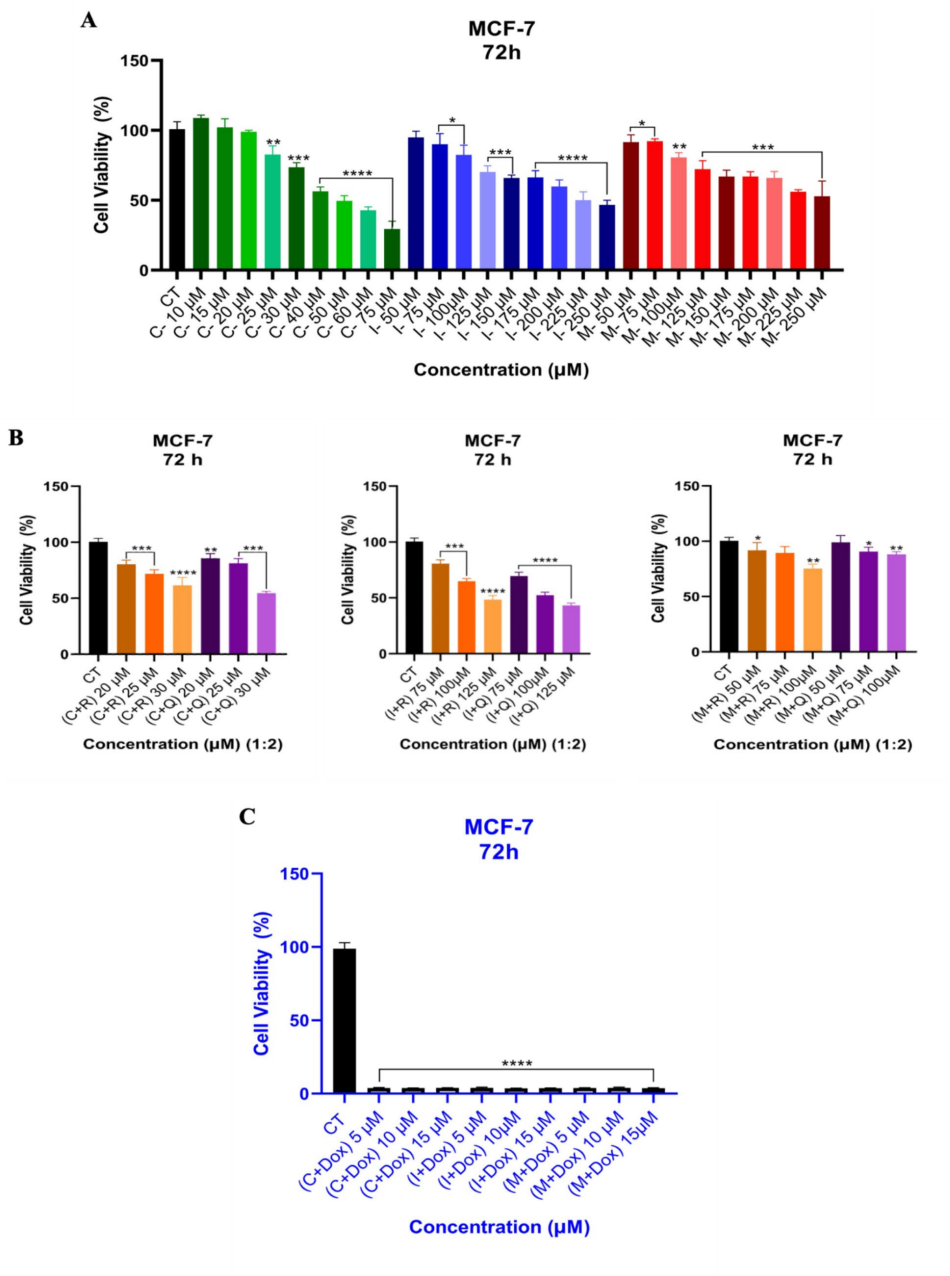
The cytotoxicity of drugs alone (**a**) and in combination with natural products (**R, Q**, and **T**), 20 µM, 25 µM, and 30 µM for drug **C**; 75 µM, 100 µM, and 125 µM for drug **I**; and 50 µM, 75 µM, and 100 µM for drug **M**. The 1:2 ratio represents drug (1) to natural product (2). **R** (20 µM), **Q** (10 µM) (**b**), and Dox (0.05 µM) (**c**) against MCF-7 ER + cells. ****p value < 0.0001, ***p value < 0.0002

As seen in Fig. 3a, drugs (**C, I, M**) alone were applied to MCF-7 cells at increasing concentrations. Although the cell cytotoxicity was obtained at 40–75 µM with drug **C**, the combination with **Q** decreased the inhibition to 30 µM. However, it was not observed inhibition below 50% in combination with **R** (Fig. 3b). The treatment with drug **I** alone showed a significant inhibition effect at a 250 µM concentration. Even though the combination with **R** showed inhibition at 125 µM, the combination of drug **I** with natural products **R** and **Q** displayed higher inhibition at 125 µM (Fig. 3b). Overall, the combination of drugs (**C, I, M**) with Dox displayed a noticeable decrease in cell viability in MCF-7 cells for combination therapy (Fig. 3c).

**Fig. 4:**
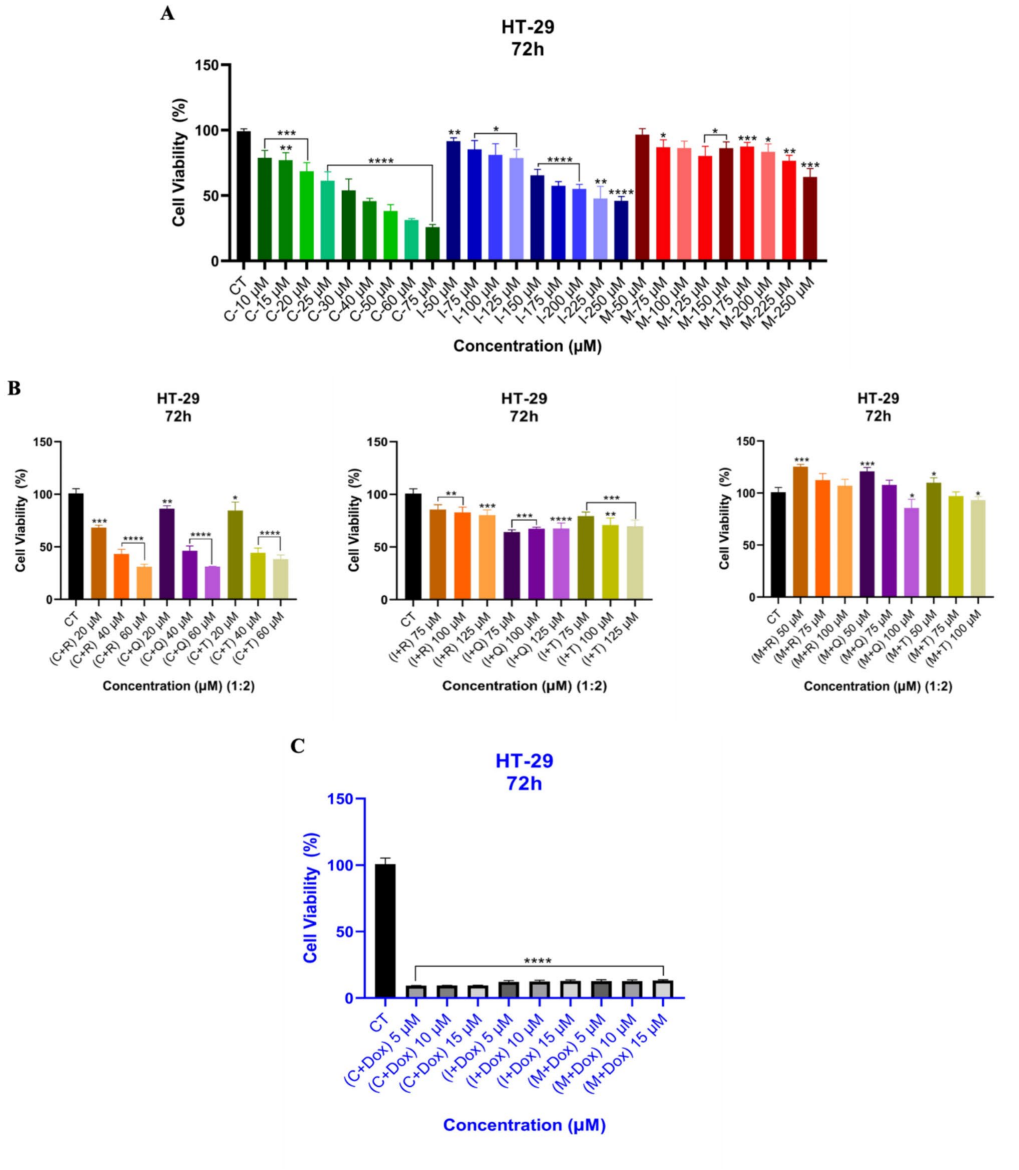
The cytotoxicity of drugs alone (**a**) and in combination with natural products (**R, Q**, and **T**), 20 µM, 40 µM, and 60 µM for drug **C**; 75 µM, 100 µM, and 125 µM for drug **I**; and 50 µM, 75 µM, and 100 µM for drug **M**. The 1:2 ratio represents drug (1) to natural product (2). **R** (20 µM), **Q** (10 µM), and **T** (10 µM) (**b**) and Dox (0.05 µM) (**c**) against HT-29 cells. ****p value < 0.0001, **p value < 0.0093

As seen in Fig. 4, drugs **C** and **I** alone inhibited the cell viability of HT-29 cells between 40 µM and 75 µM, and 225 and 250 µM, respectively. However, the cell viability was decreased at 40 µM and 60 µM, respectively, by the combination with **R, Q**, and **T**, similar to alone drug **C**. Overall, the combination of drugs (**C, I, M**) with Dox displayed a noticeable decrease in cell viability in HT-29 cells for combination therapy (Fig. 4c).

As a result, these findings highlight that the combinations of the studied drugs (**C** and **I**) with natural products and Dox increased their therapeutic efficacy on breast and colon cancer cells.

### In vivo antiulcer study

#### Comparative analysis of histopathological data using non-parametric tests

**Fig. 5:**
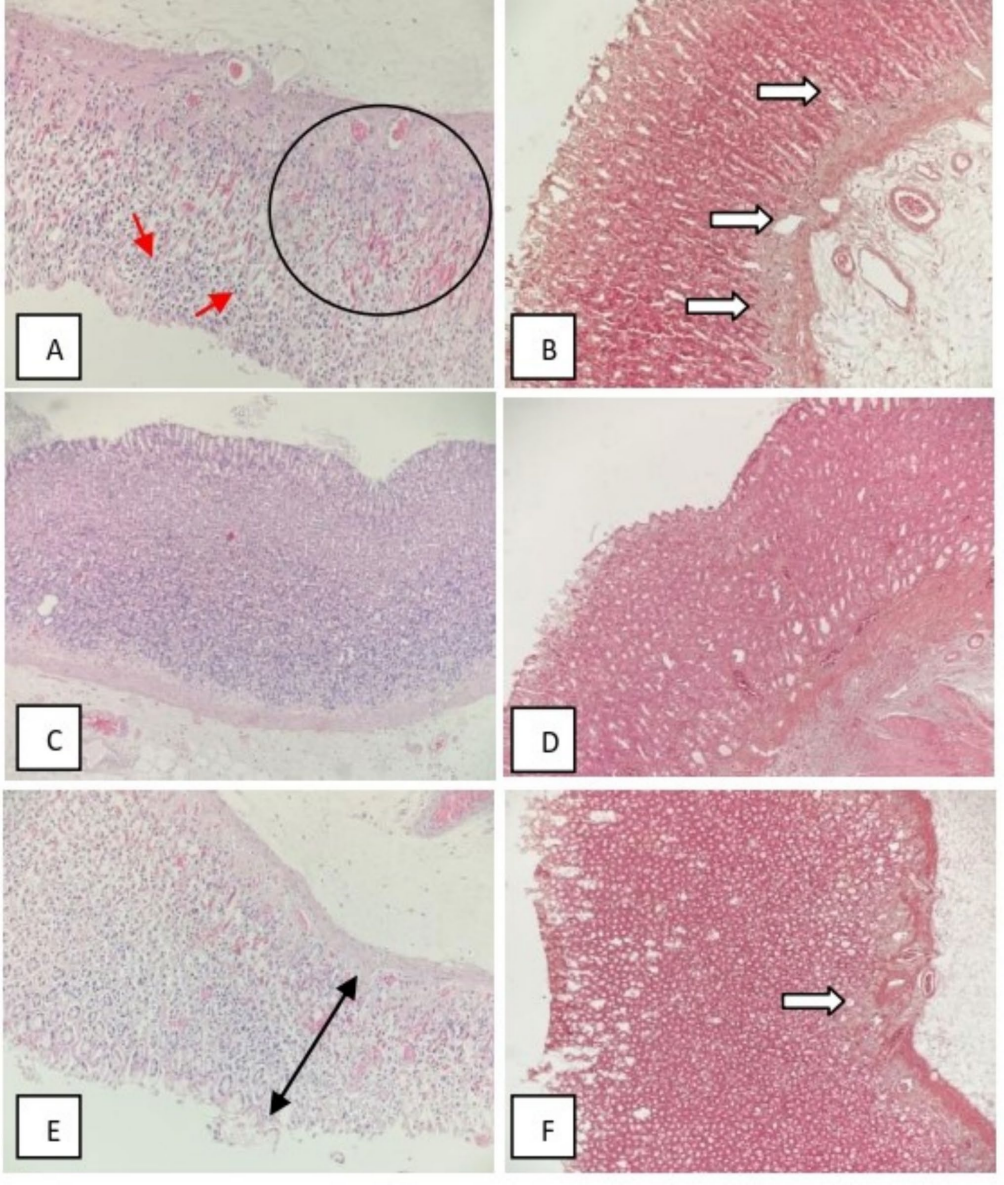
Microscopic images of UCR, UCT, and ulcer groups. **A** Neutrophil and mononuclear cell-rich appearance within the gastric mucosa, hyperemia/hemorrhage (H&E × 200). **B** Moderate to intense increased staining in the direction of fibrosis (Mason trichrome × 100). **C** A section from the UCT group showed mild inflammation, but no hyperemia/hemorrhage, erosion, or ulceration was observed (H&E × 100). **D** Mason trichrome stain did not show any staining consistent with fibrosis (Mason trichrome × 100). **E** Full-thickness ulceration was observed in the mucosal section belonging to the ulcer group (H&E × 200***). **F** Mason trichrome staining showed mild fibrosis (Mason trichrome × 100)

The Kruskal–Wallis H test was employed to assess the differences in histopathological parameters across three distinct experimental groups (Fig. 5):

$$Ulcer (U), ulcer + celecoxib (C) + rutin (UCR), and ulcer + celecoxib + taxifolin (UCT)$$

The Kruskal–Wallis test revealed statistically significant differences between the three groups for several key histopathological features. Following the observation of these overall significant differences, the Bonferroni post hoc test was applied for pairwise comparisons to isolate the specific group differences. This analysis indicated that the UCT and UCR groups were the primary contributors to the overall significance for all five parameters, demonstrating a statistically significant difference between these two treatment regimens (Table 1).

**Table 1:** Comparative analysis of histopathological data using non-parametric tests

Parameter	*p* value	*p* value (UCT vs. UCR)
Inflammation degree	0.005	0.003
Mononuclear cell infiltration	0.003	0.003
Neutrophil infiltration	0.004	0.003
Ulceration	0.006	0.005
Fibrosis	0.015	0.014

The mean rank values obtained from the Kruskal–Wallis test provide insight into the relative severity of these parameters across the groups.^1^ For all five statistically significant parameters, the UCR group consistently exhibited the highest mean rank, indicating a significantly greater severity (or degree) compared to both the U and UCT groups.

- **Inflammation degree:** UCR (mean rank = 15.71) was significantly higher than U (mean rank = 11.21) and UCT (mean rank = 6.07).
- **Mononuclear cell infiltration:** UCR (mean rank = 16.43) was significantly higher than U (mean rank = 9.57) and UCT (mean rank = 7.00).
- **Neutrophil infiltration:** UCR (mean rank = 16.50) was significantly higher than U (mean rank = 10.07) and UCT (mean rank = 6.43).
- **Ulceration:** UCR (mean rank = 15.36) was significantly higher than U (mean rank = 12.43) and UCT (mean rank = 5.21).
- **Fibrosis:** UCR (mean rank = 15.14) was significantly higher than U (mean rank = 12.07) and UCT (mean rank = 5.79).

Conversely, the Kruskal–Wallis H test demonstrated no statistically significant differences between the groups for the following histopathological features:

- Hyperemia/hemorrhage (*p* = 0.179)
- Erosion (*p* = 0.134)
- Subepithelial edema (*p* = 0.073)

In summary, the combined treatment with drug **C** and natural product **T** (UCT) appears to be associated with significantly lower histopathological severity in core inflammatory and tissue damage markers (inflammation, cell infiltration, ulceration, and fibrosis) compared to the combination with rutin (UCR).

The current results, evaluated using the Kruskal–Wallis H test and subsequent Bonferroni post hoc analysis, clearly elucidate the effectiveness of the therapeutic interventions based on the histopathological differences between the experimental groups. Statistically significant differences (*p* < 0.05) were identified in key markers of damage, including inflammation, mononuclear cell infiltration, neutrophil infiltration, ulceration, and fibrosis. The post hoc analysis determined that this significant variation primarily stemmed from the comparison between the (UCT) and (UCR) groups. This finding highlights the divergent effects of the two flavonoids (**R** and** T**) when combined with celecoxib on ulcer healing. Notably, the UCR group consistently exhibited the highest mean rank values across all these parameters, suggesting that this combination was less effective at mitigating the severity of damage compared to the other groups, and thus, the most severe histopathological profile was observed in the UCR group.

In contrast, the UCT group (**C** and** T**) presented the lowest mean rank values for all core damage markers analyzed, indicating that natural product **T** demonstrates a superior therapeutic effect in alleviating gastric ulcer injury compared to natural product **R**. This result suggests that taxifolin’s anti-inflammatory and tissue-protective properties create a synergistic effect when combined with drug **C**, significantly supporting ulcer resolution. Conversely, the absence of a statistically significant difference between the groups for hyperemia/hemorrhage, erosion, and subepithelial edema (*p* > 0.05) implies that the treatments had similar effects on these specific vascular and early-stage damage indicators, or that the variation in these parameters was insufficient to achieve statistical power. Overall, these data underscore the excellent antiulcer potential of natural product **T**, positioning this molecule as a promising combination option with drug **C** for ulcer therapy.

### In silico analysis

**Fig. 6:**
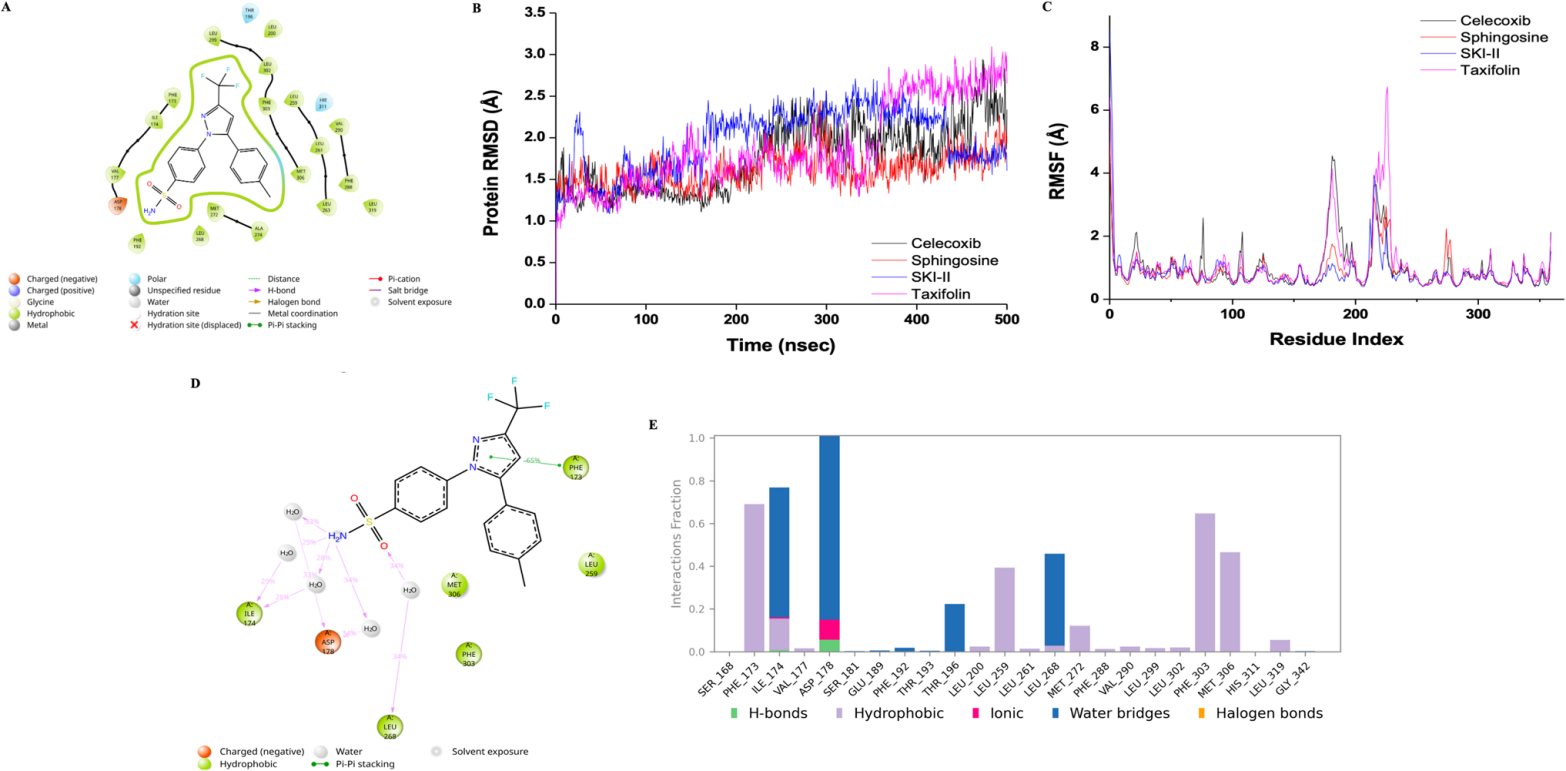
**A** 2D ligand interaction diagram of drug **C**. **B**, **C** The RMSD and RMSF graphs. **D** Ligand–protein contacts of drug **C**. **E** Protein–ligand contacts of drug **C**

In recent years, the discovery of new therapeutics targeting SphK1 has increased due to its important role in cancer metastasis (Zhan et al. 2025). Sphingolipids, lipid signaling molecules, contribute to the regulation of various cellular processes (Wang et al. 2013). In this study, the drug** C** was investigated by in silico analysis, including molecular docking and MD simulations, for the SphK1 compared with lipid substrate and known inhibitor SKI-II (Fig. 6). The drug **C** (− 8.839 kcal/mol) and natural product **T** (− 8.040 kcal/mol) showed the highest binding abilities against the SphK1. Two-dimensional (2D) ligand interaction diagram of drug **C** in the substrate binding site of SphK1 is shown in Fig. 6a. Throughout 500 ns MD simulations, the stability and conformational dynamics of the protein–ligand complexes were determined by analyzing the average root-mean-square deviation (RMSD) and root-mean-square fluctuation (RMSF). The average RMSD values with standard deviation were calculated for all complexes. The average RMSD values of drug **C**, natural product **T**, and two references (SKI-II and sphingosine) were calculated to be 1.76 ± 0.42, 1.88 ± 0.52, 1.97 ± 0.38, and 1.61 ± 0.21 Å, respectively (Fig. 6b). Moreover, the RMSF graph showed the flexibility of the residues. The RMSF values of the complexes were below 2.5 Å; however, the fluctuations in the specific residues for drug **C** and all ligands were observed above 4 Å and approximately 4 Å, respectively (Fig. 6c). Ligand–protein and protein–ligand contacts of drug **C** were shown (Fig. 6d, e). 1H-Pyrazole moiety of drug **C** displayed a pi-pi stacking with the residue Phe173, and the amino group interacted with the residues Ile174 and Asp178 (Fig. 6d). Figure 6e shows that hydrogen bonds with Ile174 and Asp178, and dominant hydrophobic interactions with the residues Phe173, Phe303, and Met306. The ionic interactions with Asp178 and Ile174 via water bridges are observed in Fig. 6e. Drugs **M** and **I** and natural product **R** have lower binding energies (< 8 kcal/mol) for SphK1 than drug **C,** natural product **T**, SKI-II, and sphingosine. Therefore, MD simulation analysis was performed for potential candidates.

## Discussion

Cancer is the most fatal disease that affects people worldwide. In this study, the potential of anti-inflammatory drugs and their natural product combination treatments for use in cancer treatment was investigated. The previously reported studies have shown that the natural product, rutin, alone or in combination with other therapeutic agents, inhibits signaling pathways, processes of carcinogenesis, and the induction of apoptosis. Additionally, rutin synergistically induces apoptosis with the therapeutic agent. It has been reported that rutin may be a promising candidate alone or in combination with other antitumor drugs for cancer treatment (Satari et al. 2021). For example, sorafenib is used to treat advanced hepatocellular carcinoma (HCC); however, drug resistance is a major problem with sorafenib therapy. In a study, the combination of quercetin with sorafenib was reported (Abdu et al. 2022).

Moreover, in a study conducted with MDA-MB-231 cells, it was reported that celecoxib caused a significant decrease in aromatase expression and cell proliferation by suppressing COX-2 (Generali et al. 2014). In a study, the treatment of celecoxib (500–1.500 mg/mL kg diet) has been concluded to significantly reduce incidence, proliferation, and tumor volume in various animal models of breast cancer (Khang et al. 2006; Khafaga et al. 2021; Van Wijngaarden et al. 2007). It was reported that the combination with curcumin and a drug for 72 h treatment inhibited the MDA-MB-231 cells’ growth in a dose-dependent manner study (10–25 µM) (Alqahtani et al. 2021). In the recently reported studies, the anticancer activities of quercetin have been identified, such as cell signaling, pro-apoptotic, antiproliferative and antioxidant effects, and growth suppression, and its potential synergistic effects with chemotherapeutic agents or radiotherapy for combination therapy (Brito et al. 2015). There are preclinical and clinical studies conducted with the combinations of quercetin with diclofenac and glucosamine. The data obtained determine the suitability of new and highly effective combinations of quercetin in medical applications (Shebeko et al. 2018). The combination of rutin with other chemotherapy drugs may provide benefits to obstruct the tumor cells and side effects of chemotherapy (Satari et al. 2021).

When the protective effect of quercetin against indomethacin’s kidney injury was investigated, the effect of quercetin on the injury of human embryonic kidney 293 cells treated with indomethacin was examined with dose- and time-dependent cell viability studies (Chen et al. 2021). Meloxicam has also been shown to induce autophagy by increasing beclin-1 and light chain 3-II (Sharpe et al. 2000).

The latest studies highlighted the potential of using celecoxib, a selective COX-2 inhibitor, in combination with traditional chemotherapeutic agents to enhance their efficacy against various cancer cell lines. It was demonstrated that the combination of celecoxib with cisplatin, paclitaxel, and Dox increased their effectiveness against human cervix cancer cells (Robledo-Cadena et al. 2020). This study also extended the investigation to SiHa cells to assess the impact on cellular growth and OxPhos flux. Similarly, it was found that celecoxib could inhibit the epithelial-to-mesenchymal transition in bladder cancer, indicating its potential in regulating gene expression and neoplastic invasiveness (Liu et al. 2019). Moreover, the studies have highlighted the cancers where celecoxib has been found as effective, such as breast cancer, non-small cell lung cancer, and hepatic carcinoma (Wen et al. 2020; Misiura et al. 2021). Additionally, it has been demonstrated that the efficacy of celecoxib against hepatocellular carcinoma is through the suppression of the COX-2/AKT/FASN cascade, further supporting the potential of celecoxib in combating liver neoplasms (Qiu et al. 2018).

Prostaglandins also suppress antitumor immunity, facilitating the invasion and metastasis of cancer cells. Celecoxib is involved in the inhibition of this proliferation and apoptosis induction (Generali et al. 2014). In fact, celecoxib does not show only a COX-2-dependent antiproliferative effect on breast cancer cells. Furthermore, indomethacin has demonstrated significant effects to inhibit cancer cell proliferation and induce apoptosis in various cancer cells (Thiruchenthooran et al. 2023). In addition, it has been reported that low-dose indomethacin accelerates apoptosis and inhibits cell proliferation in HT-29 cells (Guo et al. 2013). Additionally, it has been shown to dose-dependently activate carbonic anhydrase enzymes (I and II) and act as a mitochondrial uncoupler by regulating *β*-catenin/TCF signaling in colorectal cancer cells (Zhang et al. 2010). It was reported that the treatment of human mammary epithelial cells (HMEC) with the NSAID agent indomethacin also reduced cell invasion (Chen et al. 2021).

It is highlighted that indomethacin affects mitochondrial dynamics, leading to apoptosis in gastric cancer cells (Mazumder et al. 2019). Also, it demonstrated that indomethacin induces apoptosis in colon cancer cells by inhibiting inflammatory molecules (Seetha et al. 2020). It is found that a chimeric small molecule containing indomethacin exhibited significant cytotoxic effects on cervical cancer cells (Mishra et al. 2023). On the other hand, the studies showed that indomethacin disrupts autophagic flux in gastric cancer cells, increasing their sensitivity to cytotoxic drugs (Vallecillo-Hernández et al. 2018). The investigations have shown the protective effect of indomethacin-loaded nanoparticles against stress-induced cytotoxicity in breast adenocarcinoma cells (Franco et al. 2022). Moreover, it has been studied for its potential synergistic effects with the combinatory potential of indomethacin with polyamine-inhibitor drugs in lung cancer cell lines (López-Contreras et al. 2020).

The studies on meloxicam have shown that COX-2, which is expressed at high levels in HCC cells, inhibits the migration, adhesion, and colony formation ability of cells by increasing E-cadherin expression and decreasing matrix metalloproteinase (MMP)−2 expression (Dong et al. 2014). In addition, meloxicam has been shown to inhibit the phosphorylation of AKT, increasing the amount of pro-apoptotic proteins, including Bax and Fas-L, and inducing cell apoptosis by reducing the amount of anti-apoptotic proteins. The addition of PEG2, the major product of COX-2, can abolish the effects of meloxicam on survival and Mcl-1 expressions while not affecting Bax and Fas-L, indicating that meloxicam is both COX-2 dependent and shows that it is independently involved in cell apoptosis (Sharpe et al. 2000).

Disorders that occur in the gastrointestinal system can lead to serious health issues. One of the gastrointestinal diseases is ulcer disease, which is quite serious and reduces individuals’ quality of life. The factors such as anti-inflammatory drugs, alcohol, stress, and *Helicobacter pylori* infections may lead to ulceration (Tarnawski 2005). NSAIDs contribute to ulceration mainly in two different ways, such as topical irritation of the mucosa and the suppression of prostaglandin (PG) synthase activity produced by COX enzymes. Like all NSAIDs, celecoxib also has risks of serious gastrointestinal problems such as ulceration, bleeding, and perforation of the stomach and intestines (Dean et al., 2016; García-Rayado et al. 2018). Indomethacin is a nonselective COX inhibitor and inhibits the synthesis of PGE2 (Pacifici 2013; Lucas 2016). The increasing incidence of stomach ulcers has increased the need for new gastroprotective compounds. Natural products with significant pharmacological activities have been recommended due to their efficacy and safety (Liu et al. 2015; Athaydes et al. 2019; Ahmed et al. 2021; Neamatallah 2024). Based on its gastrointestinal effects, there are numerous studies showing that indomethacin-induced gastric ulcers occur in rats. In a recent study, caffeic acid phenethyl ester (CAPE), which is a natural bioactive compound, prevented indomethacin-induced gastric ulcer in rats (Neamatallah 2024). Compared to standard NSAIDs, meloxicam tenfold selectively inhibits COX-2 over COX-1. On the other hand, flavonoids protect the mucosa by showing antioxidant, anti-secretory, and cytoprotective properties (Zayachkivska et al. 2005; Martin et al. 1994). As a result of a reported study, it has been announced that the gastroprotective activity of *A. aspera* may be due to the presence of flavonoids and tannins (Das et al. 2012). Another similar study conducted on the antiulcer activity of *Ficus religiosa* leaf extract. Stress-induced ulcers in rats have been examined. The antiulcer activity of this plant leaf extract is probably due to the presence of flavonoids (Gregory et al. 2013).

Quercetin is a flavonoid, found in many fruits and vegetables, that also has lots of potential for anticancer, anti-inflammatory, antidiabetic activities, etc. (Sayeed et al. 2017; Chen et al. 2016; Kashyap et al. 2016). Taxifolin, also known as dihydroquercetin, is a subclass of flavanonols in the flavonoids family. It has a broad spectrum of pharmacological activities such as anti-inflammatory, antiangiogenic, and chemotherapeutic properties. Taxifolin is also associated with the inhibition of the expression of COX-2 and PGE2 (Wang et al. 2006; Oi et al. 2012). Piceatannol (PIC), a natural polyphenolic stilbene, exhibited potential gastroprotective effects against indomethacin-induced gastric ulcers via its antioxidant, anti-inflammatory, and angiogenic properties (Shaik and Eid 2022). Adult male Wistar rats were used and divided into five groups such as control group, indomethacin group, Indo + PIC group, and Indo + omeprazole group, in which omeprazole is used to treat elevated acid levels in the stomach (Shaik and Eid 2022). In another study, the protective properties of *Apium graveolens* L. seed extract against indomethacin-induced ulceration were examined in rats. Male Wistar rats were divided into four groups. The control group received only carboxy methylcellulose (CMC) for 7 days. The ulcer group received indomethacin after CMC treatment. It demonstrated the gastroprotective effects of the extract, which include reducing gastric acidity, restoration of normal mucosa, and reduction of the degenerative alterations in stomach glands (Abu-Baih et al. 2024). Furthermore, tetramethylpyrazine (TMP) was used against indomethacin-induced gastric ulcer in rats. Forty-two adult male Wistar rats were used and divided into six groups. In conclusion, the gastroprotective effects of TMP against indomethacin-induced gastric ulcer were reported (AlKreathy et al. 2020). Additionally, it was reported regarding the most effective doses of omeprazole, misoprostol, and celecoxib in ulceration. It showed the most effective ulcer healing. Celecoxib postponed COX-2 expression and delayed ulcer healing (Poonam et al. 2005). Consequently, this study’s findings may highlight the usage of celecoxib as an antiulcer and anticancer agent in combination with taxifolin.

Lipid–protein interactions between sphingosine and SphK1 were shown in a reported study (Wang et al. 2013). The hydroxy group of sphingosine interacted with Asp178. SphK1 inhibitor SKI-II binding in the lipid-binding pocket of SphK1 with overlaid sphingosine lipid was shown. The hydroxy moiety, an amino group, and a chlorine atom interacted with the residues, including Asp178, Thr196, and Phe288, to form hydrogen bonds (Wang et al. 2013). The rest of SKI-II forms van der Waals interactions with the hydrophobic tail of the lipid substrate. As a result, it is reported that SKI-II is a lipid substrate competitive inhibitor (Wang et al. 2013). It is clear that specific and potent SphK1 inhibitors play an important role in the treatment of cancer (Yi et al. 2023). Although many SphK1 inhibitors and/or potential inhibitor candidates have been reported, click products for the first time using computational approaches were investigated (Allıto et al. 2025a, b); Erdoğan et al. 2024; Erdoğan and Comert Onder, 2024). In previously reported studies, the binding affinities of the known SphK1 inhibitor SKI-II were reported to be − 8.8 kcal/mol (Jairajpuri et al. 2020). In a reported study, naturally occurring compounds were screened for targeting SphK1. A hydrogen bonding interaction was reported with the substrate binding residue Asp178 of the target (Önder et al. 2024).

## Conclusion

In conclusion, the histopathological analysis demonstrates that the combined treatment of UCT is associated with a significantly attenuated severity of inflammatory and tissue damage markers. Specifically, the UCT regimen resulted in lower mean ranks for inflammation, mononuclear cell infiltration, neutrophil infiltration, ulceration, and fibrosis, indicating a superior therapeutic effect compared to the combined treatment of UCR. This suggests that the substitution of rutin with taxifolin, when combined with celecoxib, leads to a statistically significant improvement in the amelioration of gastric mucosal injury. In silico studies make it possible to reposition existing drugs on the market and pioneer in vitro and in vivo research. Therefore, computer-aided studies are increasingly important for identifying promising drug candidates. In silico approaches are generally less expensive and faster to implement than experimental drug discovery methods. Thus, in silico methods are of great importance in target identification and the prediction of new drugs (Pushpakom et al. 2019). In this study, in silico analyses highlight that drug **C** and natural product** T** may be potential inhibitor candidates targeting SphK1-based cancer therapy. It was concluded that drug **C** could guide the design of new therapeutic candidates or other cancer drug targets for future studies.

## Data Availability

The data that support the findings of this study are available from the corresponding author upon reasonable request.
